# Activity of trastuzumab emtansine (T-DM1) in 3D cell culture

**DOI:** 10.1007/s10549-021-06272-x

**Published:** 2021-06-05

**Authors:** Jean Zheng Boyer, Gail D. Lewis Phillips, Hiro Nitta, Karl Garsha, Brittany Admire, Robert Kraft, Eslie Dennis, Elizabeth Vela, Penny Towne

**Affiliations:** 1 Roche Tissue Diagnostics, 1910 E Innovation Park Drive, Tucson, AZ 85755 USA; 2 Genentech, Inc., South San Francisco, CA USA

**Keywords:** Breast cancer cell lines, 3D cell culture, T-DM1, Drug efficacy study, Heterogeneity, Drug resistance

## Abstract

**Background:**

Cell spheroids and aggregates generated from three-dimensional (3D) cell culture methods are similar to in vivo tumors in terms of tissue morphology, biology, and gene expression, unlike cells grown in 2D cell cultures. Breast cancer heterogeneity is one of the main drug resistant mechanisms and needs to be overcome in order to increase the efficacy of drug activity in its treatments.

**Methods:**

We performed a unique 3D cell culture and drug efficacy study with trastuzumab emtansine (Kadcyla®, T-DM1) across five breast cancer cell lines (BT-474, SK-BR-3, MDA-MB-361, MDA-MB-175, and MCF-7) that were previously investigated in 2D cell culture. We performed HER2 IHC staining, cell viability experiments, Gene-protein-assay (GPA), and T-DM1 internalization studies.

**Results:**

We obtained significantly different results including higher IC_50_ for some of the cell lines. Our GPA showed some significant heterogeneous *HER2* gene and protein expression in 3D cultured spheroids or aggregates. The fluorescent images also showed that a longer incubation time is needed for T-DM1 to be internalized effectively into 3D cultured spheroids or aggregates.

**Conclusion:**

Our study demonstrated that the difference of T-DM1 drug activity in 3D spheroids or aggregates might be due to tumor heterogeneity and less efficient internalization of T-DM1 that is not seen using 2D cell culture models. Drug studies using 3D cell culture are expected to provide biologically relevant models for determining drug activity in tumor tissue in future drug response and resistance research.

## Introduction

Despite significant medical advances, breast cancer remains the leading cause of cancer and second leading cause of cancer related death in women [1]. Breast cancer heterogeneity results from genome mutations, gene expression levels, tumor immune status, the microenvironment, etcetera [2–5]. Intratumor heterogeneity could be one of the main resistance mechanisms of breast cancer therapy [6–8].

The *HER2* gene encodes a transmembrane receptor of the epidermal growth factor family of receptor tyrosine kinases. Amplification of the *HER2* gene occurs in about 15–20% of breast cancers and leads to proliferation, angiogenesis, and invasiveness of neoplasms [9]. Multiple tumor cell subpopulations with varying *HER2* gene amplification and/or expression levels of HER2 protein within the same tumor defines intratumor heterogeneity [10, 11]. The prevalence of HER2 heterogeneity is reported in 30% of HER2 positive patients [10–12]. Intratumor heterogeneity could reduce drug efficacy and be an independent factor for resistance to anti-HER2-targeted therapy [10, 13].

It would be beneficial to reproduce intratumor heterogeneity using cell culture models in order to develop new targets for drug discovery, testing, and development. However, the phenomenon of intratumor heterogeneity is extremely difficult to reproduce using traditional 2D cell culture methods. Cells derived from tumor tissue and grown using 2D cell culture do not form the multidimensional, 3D structure of a tumor, whereas 3D cell culture methods are better (although not exact) models of 3D in vivo tumors or tissues [14]. Comparison of 2D and 3D include loss of epithelial cell polarity and altered epithelial and fibroblast shape in 2D, with cells in 2D versus 3D having different patterns of gene expression, as well as differences in other biological functions [14]. 2D and 3D cell culture models would typically be used during pre-clinical/translational research and drug discovery studies. Targets identified using a 2D approach may fail during clinical trials because the data from 2D models may not reflect in vivo patient tumors [15]. Animal models frequently provide definitive tests of specific molecules and processes in translational research [14]. In vitro 3D cell culture models provide an approach that bridges the gap between traditional 2D cell culture models and animal models, and reduce the number of animals used in tumor research and drug evaluation.

When 3D cell culture methods are applied, the cultured cells may form spheroids or aggregates that mimic the morphology, gene expression, metabolism, and cell–cell or cell–extracellular matrix (ECM) interactions found in tumor tissues [15]. Functional cellular heterogeneity results from the complex cellular composition and differential gene expression within spheroids or aggregates. A 3D spheroid contains zones that include a proliferative outer layer, a quiescent inner layer, and sometimes a necrotic center. The cells in the outer proliferative layer have easy access to oxygen, nutrients, and growth factors; these cells maintain cell cycles and undergo cell division [16]. The middle, quiescent cell layer resides where oxygen and nutrients are less available; hence, the cells are viable but undergo cell cycle arrest and are in a dormant or quiescent state [16]. The center of spheroids may contain a necrotic zone of cells that died due to insufficient oxygen and nutrients and accumulated waste. Consequently, the surface biomarker, cell–cell, cell–ECM, metabolism, and drug response dependent intracellular signaling pathways may be different in 3D cultured spheroids and aggregates when compared with 2D cultured monolayers [17, 18]. Therefore, intratumor and/or functional cellular heterogeneity in 3D cultured cancer spheroids and aggregates holds promise as a convenient means to mimic the biologically relevant features of tumors and tissues found in cancer patients that may affect drug penetration, internalization, efficacy, and drug resistance.

Trastuzumab emtansine (T-DM1) is an antibody–drug conjugate (ADC) approved as the second line of treatment for HER2 positive metastatic breast cancer. Trastuzumab binds to overexpressed HER2 receptors on cell surfaces where the *HER2* gene is amplified. Subsequently, T-DM1 is internalized into HER2 positive cancer cells where lysosomal degradation leads to release of the active chemotherapy DM1 from the trastuzumab. DM1 containing cytotoxic catabolites prevent microtubule polymerization which in turn inhibits cell division and leads to cell death during the mitotic stage [19].

We performed a drug efficacy study with 3D cell culture and T-DM1 across five breast cancer cell lines that were previously investigated with 2D cell culture [20]. We measured the response of cancer cells to T-DM1 when grown as 3D spheroids or aggregates and as a 2D monolayer. We used a HER2 GPA method [21] to detect heterogeneity in 3D cultured spheroids or aggregates. A feature of our study is to observe how heterogeneity, in 3D spheroids or aggregates affects drug efficacy by influencing T-DM1 internalization and cell death.

## Materials and methods

### T-DM1 antibody–drug conjugate

Stock solutions (20 mg/mL) of T-DM1 (Genentech, South San Francisco, CA) were prepared in cell culture quality distilled water and stored at − 80 °C.

### Cell lines and reagents

Breast cancer cell lines BT-474, SK-BR-3, MDA-MB-361, MDA-MB-175. MCF-7 and their growth media were purchased from American Type Cell Collection (ATCC, Manassas, VA) and grown according to specifications.

### Immunohistochemistry (IHC) assay for 3D and 2D cultured cells

Formalin-fixed, paraffin-embedded (FFPE) uniblocks were prepared with 2D cultured monolayers (traditional methods) and 3D cultured spheroids or aggregates (novel methods) for each cell line.

For 3D cultured cells, each cell line was initially cultured in 2D flasks to a total cell population of 3 × 10^7^. Cells were then transferred to Nunclon™ Sphera™ flasks (Thermo Fisher Scientific, Waltham, MA) and grown for an additional 3–4 days until 3D spheroids or aggregates formed. The cells collected from 2D cultured monolayers or 3D cultured spheroids or aggregates were washed with Hanks’ Balanced Salt Solution (#14025, Life Technologies, Carlsbad, California) and fixed in 10% neutral buffered formalin. Cell pellets were mixed with HistoGel™ (HG-4000-012, Thermo Fisher Scientific) followed by cell processing and paraffin embedding.

Several 4-μm slides were sectioned from each 2D or 3D paraffin block. HER2 protein is detected with 4B5 (clone) on a BenchMark ULTRA automated staining system (RTD, Tucson, AZ) in accordance with the recommended procedure. A pathologist and a qualified reader scored the stained HER2 slides based on the IHC staining intensity.

### *Cell viability assays comparing IC*_*50*_* for 2D and 3D cell culture*

For 2D cell culture, cells were seeded on a Corning™ 96-well plate (CLS3603, Corning™, MA) for 24 h at 20,000 cells/well for BT-474 and 10,000 cells/well for SK-BR-3, MDA-MB-361, MDA-MB-175 and MCF-7 [20].

For 3D cell culture, we seeded cells in 3D Corning™ ultra-low adhesion spheroid microplates (96-well) (CLS4520 Corning™, MA) at 10,000 cells/well for BT-474 and 6000 cells/well for SK-BR-3, MDA-MB-361, MDA-MB-175, and MCF-7.

After 24 h, the 2D and 3D cultured cells were treated with various concentrations of T-DM1 for 72 h. CellTiter-Glo® 3D Cell Viability Assay (G9683 Promega, Madison, WI) reagent was added into each well at the end of the 72-h drug treatment following the manufacturer’s recommendations. Luminescent signals of each well were quantified with Synergy H4 Hybrid Reader (model H4MLFA, BioTek, VT). Prism GraphPad™ software (GraphPad™ Software, San Diego, CA) was used for statistical analysis.

### HER2 GPA (gene-protein assay)

We conducted HER2 GPA for the simultaneous visualization of *HER2* gene amplification and chromosome 17 centromere (CEN17) signal as well as HER2 protein expression on the sections of FFPE 3D cultured spheroids or aggregates. The HER2 GPA study was performed as described previously [21, 22]. To evaluate the *HER2* gene signal and ratio of HER2/CEN17, black dots (*HER2* gene signal) and red dots (CEN17 signal) were manually counted for 20 cells from each slide and two separate slides for each cell line. The mean of the HER2 and CEN17 signals were calculated and used to determine the ratio of HER2/CEN17.

### T-DM1 internalization assay

Zenon™ pHrodo™ iFL Red Human IgG Labeling Reagent (Z25612; Thermo Fisher Scientific) was incubated with T-DM1 following manufacturer recommendations. We used a Molecular Probes® Cell Imaging Kit (R10477; Thermo Fisher Scientific) containing NucGreen™ reagent (dead cell indicator) and NucBlue™ reagent (a total cell indicator). We seeded BT-474, SK-BR-3, MDA-MB-361 and MCF-7 in 3D 96-well plates for 24 h. As a positive control, we also cultured BT-474 in 2D 4-well chamber slides for 24 h. We next treated the 2D and 3D cells with Zenon™ pHrodo™ labeled T-DM1 (3 μg/mL). Some of the wells were incubated for time periods of 24, 48, and 72 h. Human IgG isotype control (#31154; Thermo Fisher Scientific) (3 μg/mL) labeled with Zenon™ pHrodo™ was used as a negative control. At the end of each treatment period, one drop of NucGreen™ reagent and one drop of NucBlue™ reagent were added to each well and incubated for 60 min prior to imaging with a Zeiss Axio Imager M2 fluorescent microscope (Zeiss, Thornwood, NY). The microscope was equipped with a Photometrics CoolSnap ES2 cooled monochrome camera (Teledyne Photometrics, Tucson, AZ) and appropriate filters (Chroma Technology, Bellows Falls, VT) for separation of NucGreen™, NucBlue™ and Zenon™ pHrodo™ fluorescent signals. The excitation/emission filters for NucBlue™, NucGreen™ and Zenon™ pHrodo™ iFL were 350 ± 25 nm/460 ± 25 nm, 490 ± 10 nm/520 ± 10 nm and 580 ± 12 nm/625 ± 15 nm, respectively. Image analysis was performed with FIJI (ImageJ) software.

## Results

### HER2 protein expression status in select 3D and 2D cultured cells

**Fig. 1:**
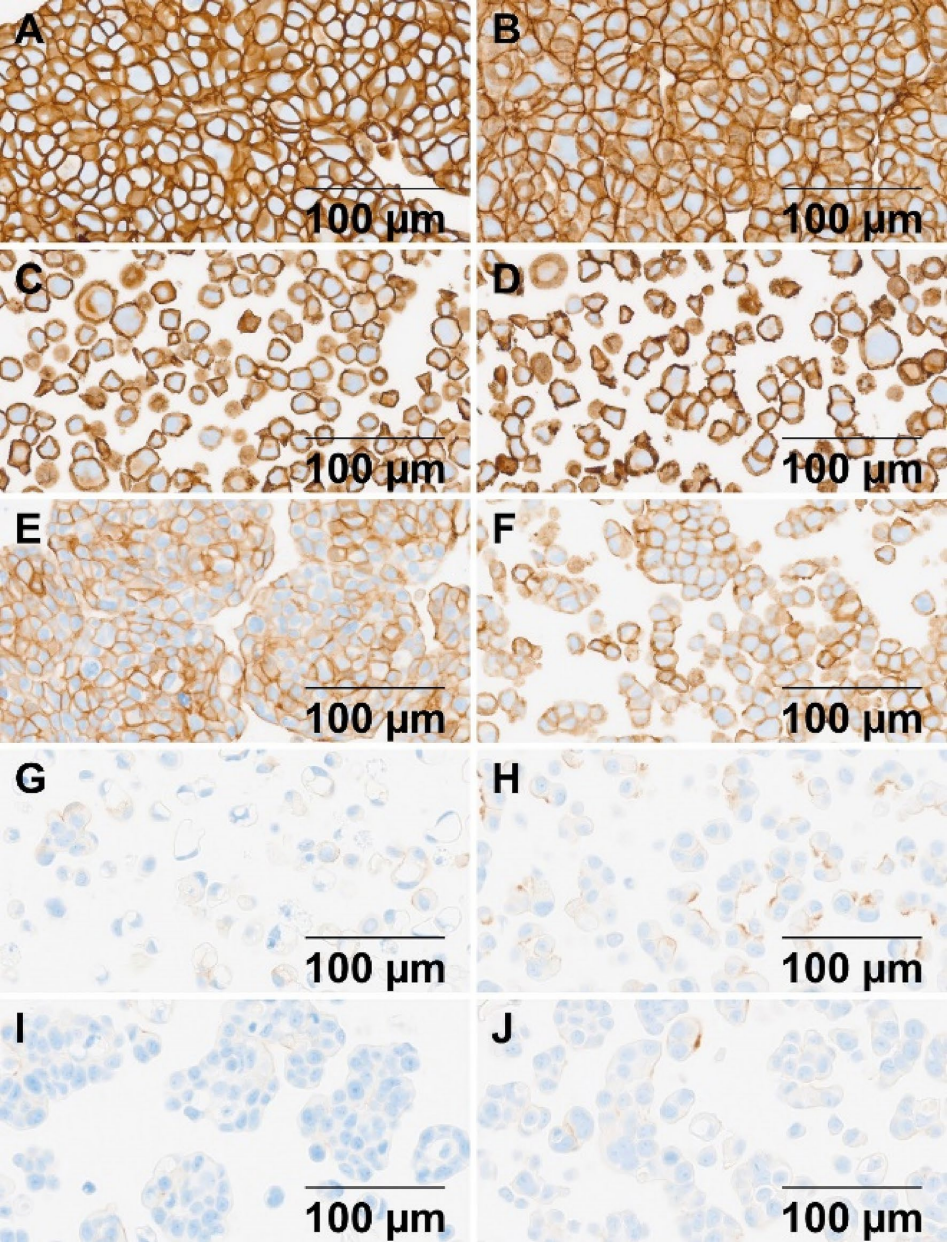
Images of HER2 protein expression levels are shown for the five cell lines grown as 3D and 2D cell cultures: **A**, **B** 3D and 2D BT-474; **C**, **D** 3D and 2D SK-BR-3; **E**, **F** 3D and 2D MDA-MB-361; **G**, **H** 3D and 2D MDA-MB-175; and **I**, **J** 3D and 2D MCF-7

Figure 1 shows HER2 protein expression (IHC staining) results for the breast cancer cell lines studied. The IHC staining intensity scores for HER2 shown in Table 1 are the same for 3D cultured spheroids or aggregates and 2D cultured cells as previously reported [23–25]. The IHC staining intensity scores indicate that the HER2 protein is highly overexpressed (3+) in BT-474 and SK-BR-3 and moderately overexpressed (2+) in MDA-MB-361. In addition, HER2 protein is modestly overexpressed in MDA-MB-175 (1+), with obvious membrane staining, while MCF-7 shows faint-to-moderate incomplete membrane staining (1+) in about 10% of the cells. Previous studies showed that HER2 is expressed in MCF-7 at the level of normal epithelial tissue (IHC score 0) [25, 26], and therefore used as the negative control. The results for HER2 protein expression level are independent of whether cells are cultured with 2D or 3D methods.

**Table 1:** Summary of viability assay and HER2 IHC staining results

Cell line	Viability assay relative IC_50_ (µg/mL)		Fold change	HER2 IHC intensity level
	2D	3D		
BT-474	0.178	6.71	37.7	3+
SK-BR-3	0.00876	0.0366	4.18	3+
MDA -MB-361	0.0912	1.99	21.8	2+
MDA-MB-175 VII	0.35	1.49	4.3	1+
MCF-7	17.3	16.8	0.974	1+

**Fig. 2:**
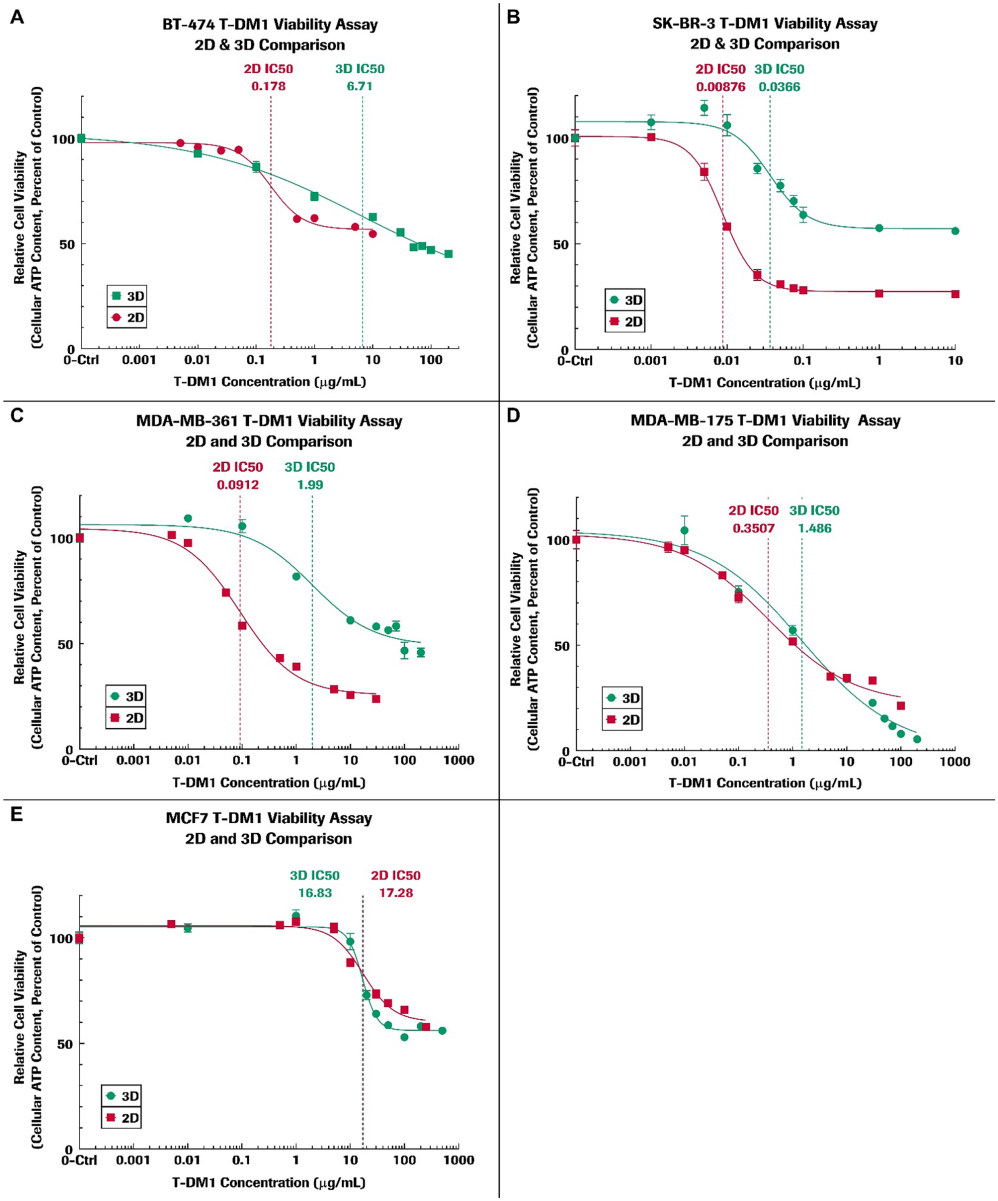
Viability assay results are shown as plots of relative cell viability versus T-DM1 concentration for BT-474, SK-BR-3, MDA-MB-361, MDA-MB-175, and MCF-7 cell lines using 2D and 3D culture methods as described in the text. The 2D cell viability results are in red and 3D results in green. GraphPad Prism software was used for statistical analysis and calculation of the best-fit curves and for plotting data. The data points on each plot are generated from six replicates and are represented on the plot as mean ± standard error of the mean. The estimated T-DM1 IC_50_ value based on the best-fit curves are labeled and shown as vertical dashed lines

The IC_50_ results from Fig. 2 are shown for the five cell lines grown as 2D and 3D cultures. The calculated IC_50_ fold change and HER2 IHC staining intensities are also shown

### Comparison of 2D and 3D viability results of cells treated with T-DM1

Figure 2 shows the plots of cell viability versus T-DM1 drug concentration and the best-fit drug response curves from which the relative IC_50_ value is obtained for each of the respective 2D and 3D cultured cell lines. Our T-DM1 drug efficacy data from 2D cultured cells successfully reproduced the previously published 2D results [20]. Table 1 summarizes the IC_50_ results and the calculated fold change in IC_50_ for the 3D spheroids or aggregates relative to 2D cells. The relative IC_50_ for 3D cultured spheroids or aggregates was frequently much higher than for 2D cultured cells. However, MCF-7 cells did not show a significant fold change in IC_50_ for 2D and 3D cultured cells and the drug response curves for 2D and 3D are similar to each other.

### HER2 gene and protein status with GPA staining in 3D spheroids or aggregates

**Fig. 3:**
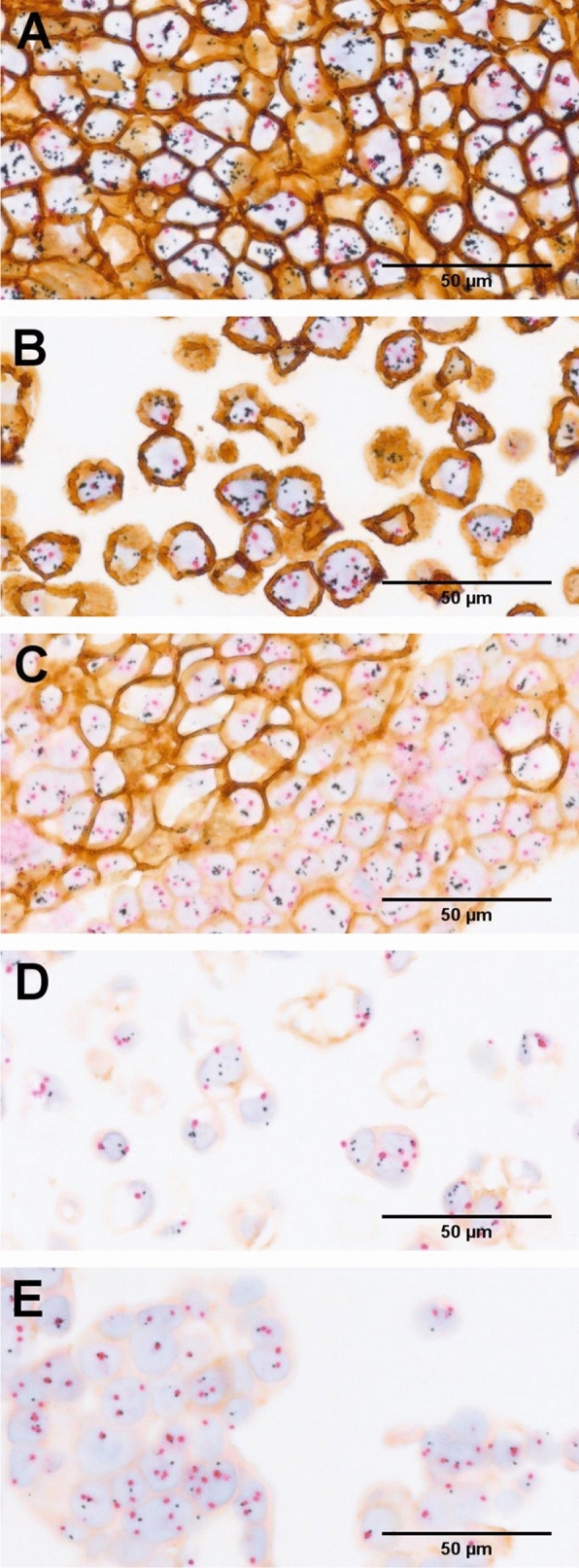
Images of GPA results displaying HER2 gene amplification and protein expression levels in 3D spheroids or aggregates are shown. The presence of the HER2 gene is indicated in the assay as small black dots. The presence of CEN17 is indicated as small red dots. The presence of the HER2 protein is indicated as brown staining. **A** 3D BT-474; **B** 3D SK-BR-3; **C** 3D MDA-MB-361; **D** 3D MDA-MB-175, and **E** 3D MCF-7

Since tumor heterogeneity is one of the major obstacles for drug effectiveness and resistance, we explored whether heterogeneous *HER2* gene amplification and HER2 protein expression levels contribute to the higher IC_50_ in 3D cultured spheroids or aggregates. We performed a GPA study to determine HER2 protein expression and *HER2* gene amplification status simultaneously in the 3D cultured cell lines. The status of the *HER2* gene (black), CEN17 (red), and HER2 protein (brown) signals is shown in Fig. 3. The HER2 GPA scoring data, obtained from the images, are summarized in Table 2.

**Table 2:** HER2 GPA scoring data

HER2 GPA scoring data										
	BT-474		SK-BR-3		MDA-MB-361		MDA-MB-175		MCF-7	
	HER2	CEN17	HER2	CEN17	HER2	CEN17	HER2	CEN17	HER2	CEN17
Mean signal per cell	15.73	3.53	13.70	4.95	8.45	3.10	3.20	2.90	2.13	3.98
Ratio (HER2/CEN17)	4.46		2.77		2.73		1.10		0.53	

The HER2 GPA scoring data obtained for the five breast cancer cell lines were manually counted from GPA HER2 images and used to determine the mean signal per cell for the HER2 gene and CEN17 from which the HER2/CEN17 ratio is calculated and displayed as shown in the table

There is no obvious heterogeneity observed between *HER2* gene and protein expression levels in either the BT-474 spheroids (Fig. 3A) or the SK-BR-3 aggregates (Fig. 3B). However, we observe heterogeneous HER2 protein expression levels within certain regions of the 3D cultured MDA-MB-361 cells (Fig. 3C). There are regions of darker brown indicating relatively higher HER2 protein expression, as well as regions of lighter or no brown color indicating relatively low HER2 protein expression level. In 3D MDA-MB-175 aggregates (Fig. 3D), the average *HER2* gene signal per cell was 3.2, the HER2/CEN17 ratio was 1.1 and the IHC intensity level of the HER2 protein was 1+. The *HER2* gene was amplified slightly in a small percentage of the MDA-MB-175 cells (Fig. 3D). However, for the 3D MCF-7 aggregates (Fig. 3E), the *HER2* gene signal per cell was 2.13, the HER2/CEN17 ratio was 0.53 and the IHC intensity level of the HER2 protein was 1+. In summary, we observed significant heterogeneous HER2 protein expression among 3D cultured MDA-MB-361 cells and *HER2* gene amplification in a small percentage of the MDA-MB-175 cell aggregates. However, we did not observe heterogeneous *HER2* gene amplification and protein expression by GPA assay in 3D cultured BT-474, SK-BR-3 or MCF-7 spheroids or aggregates.

### T-DM1 internalization into 3D spheroids or aggregates

**Fig. 4:**
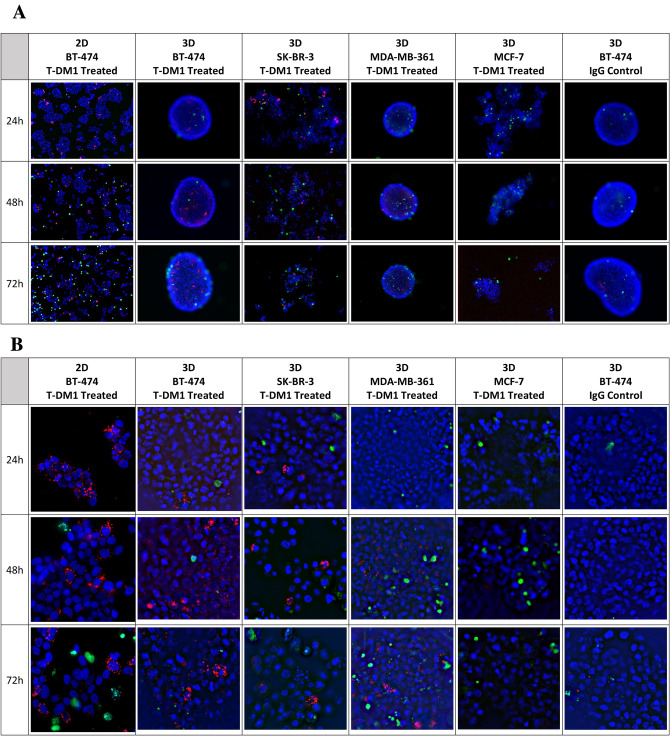
This figure shows internalization of fluorescent pHrodo™-labeled T-DM1 (red color) for cells grown as 3D spheroids or aggregates. Images in **A** are at ×8 magnification. Images in **B** are at ×20 magnification. The ×20 images of **B** are derived from the same cells, spheroids, or aggregates as the ×8 images of **A**. Dead cells are green in color (produced by NucGreen) and total cell nuclei are blue in color (produced by NucBlue). Images are arranged in rows showing a time course of T-DM1 internalization for 24 h, 48 h, and 72 h. The columns are labeled to show the cell lines and culture method used in the imaging. The first column shows the 2D BT-474 treated with pHrodo-labeled T-DM1 as a positive control, while the last column shows 3D cultured BT-474 cells treated with pHrodo-labeled IgG in BT-474 cells as a negative control

We used pHrodo™ labeled T-DM1 to observe internalization of T-DM1 in 3D cultured BT-474, SK-BR-3, MDA-MB-361 and MCF-7 cells. The pHrodo™ iFL dye is a colorless dye which converts to fluorescent red after it is internalized into the cell and reaches the lysosomes. We selected 2D cultured BT-474 cells as a positive control and 3D BT-474 cells treated with pHrodo™ labeled IgG as a negative control for our internalization study. The resulting images are shown in Fig. 4A (8×) and Fig. 4B (20×).

In the 2D cultured T-DM1 treated BT-474 cells (Fig. 4), the punctate red fluorescent signals indicate internalization of pHrodo™ labeled T-DM1 starting at about 24 h after treatment and no increase in red signal intensity beyond 24 h in the 2D culture. Internalization is at a maximum by 24 h, with many dying cells stained with green dye observed to be floating away at 48–72 h after drug treatment. The negative IgG control in 3D BT-474 shows a few red signals at 72 h that appear to be due to pinocytosis.

In contrast, for T-DM1 treated 3D (BT-474), there was minimal signal at 24 h, with increased signal at 48 and 72 h, indicating slower kinetics of internalization in 3D cultures. Some green signal is present in all images indicating dead or dying cells. We observed punctate red fluorescent signals indicating T-DM1 internalization into 3D cultured SK-BR-3 aggregates at about 24 h (Fig. 4). In addition, we observed fragmented nuclei at 72 h in SK-BR-3 (Fig. 4B), indicating that many cells were undergoing apoptosis by that time. However, minimal red fluorescent signal is visible in 3D cultured MDA-MB-361 at 24 h (Fig. 4). The red and green signals increased and became readily visible at 48 h and 72 h. MCF-7 cells did not exhibit *HER2* gene amplification although the HER2 protein expression is 1+. However, there was minimal green signal (dead cells) which may indicate non-specific uptake of T-DM1 as a result of pinocytosis (Fig. 4).

## Discussion

Our data demonstrate that relative IC_50_ values are frequently higher in 3D cultured spheroids or aggregates than for the corresponding 2D monolayers. There are also differences that may be due to the type of 3D structure that naturally occurs. For example, the BT-474 and SK-BR-3 cells show HER2 IHC intensity score of 3+ (Fig. 1). When comparing 3D and 2D fold changes in IC_50_ values, BT-474 has a fold change of about 37.7, while SK-BR-3 has a fold change in IC_50_ of 4.18. The 3D cultured BT-474 cells form very tight, cohesive spheroids displaying cell–cell adhesion [26], while 3D cultured SK-BR-3 cells form loose, grape-like structured aggregates. The differences in physical structure, such as tight spheroids versus loose aggregates, may affect T-DM1 penetration and distribution. For example, T-DM1 may not effectively penetrate physical barriers of tight and compact spheroids and subsequently binds primarily to the HER2 receptors on the outer spheroid surfaces where the T-DM1 internalizes into the cells [27]. Thereafter, the outer layer of cells on a 3D spheroid or aggregate is peeled away like an onion due to the dying cells, thus exposing more cells to the T-DM1 presented in the media. On the other hand, the physical structure of SK-BR-3 cells is a loose 3D aggregate and is therefore closer to cells grown in a 2D monolayer. The loose aggregates of 3D cultured SK-BR-3 are exposed to the T-DM1 in a manner somewhat similar to the 2D cultured cells, making it much easier to bind and internalize the T-DM1, resulting in a smaller IC_50_ fold change (Table 1). It is important to note that the drug results of 3D cell cultured spheroids and aggregates for all five cell lines are more likely to resemble patient tumors in vivo than the results from 2D cultured cells, with the exception of 3D SK-BR-3 results.

Heterogeneous *HER2* gene amplification and protein expression have been previously studied in breast cancer tissues [21, 28], but not in 3D cell cultures. Previous research has shown that 3D cultured spheroids demonstrate functional heterogeneity such as different cell cycle phases based on their location within the spheroids [16, 29]. A previous study showed that T-DM1 efficiently inhibited proliferation with cell arrest in the G2-M phase and induced cell death by apoptosis in cells with a significant level of surface expression of HER2 [30]. Another potential reason is that hypoxia and nutritional environment gradients within the structure of the 3D spheroids or aggregates cause the cells to enter various cell cycle phases or even cell cycle arrest, resulting in cells with different proliferation status and having a varied response to drugs [16, 31, 32]. The cells within a hypoxic environment in the spheroids may also present upregulated expression of hypoxia-inducible family factors and Multi Drug Resistance (MDR) gene [33, 34].

The efficacy of T-DM1 depends on binding to overexpressed HER2 receptors on cancer cell surfaces, followed by internalization, and lysosomal trafficking and catabolism. Consequently, T-DM1 has limited activity in cells with low HER2 protein expression [35] as observed in our MCF-7 cells cultured with the 2D and 3D methods. Therefore, T-DM1 has a limited effect on the cells with non-amplified HER2 receptors no matter whether the cells are grown as a 2D or 3D culture.

Drug resistance is a major obstacle in effective cancer treatment. A recent study [36] demonstrated that T-DM1 is ineffectively internalized into lysosomes because of accumulation of T-DM1 in CAV1 vesicles. Another study [37] showed that mutation of SLC46A3, a lysosomal membrane protein that transports the T-DM1 catabolite, Lys-MCC-DM1, from lysosome to cytosol, also contributed to T-DM1 drug resistance. In yet another study [38], it was concluded that cyclin B1 induction was responsible for cell sensitivity to T-DM1, while silencing of cyclin B1 resulted in resistance to T-DM1. Other factors may also affect the efficacy of T-DM1 in tumors. Furthermore, a heterogeneous cell population could contain breast cancer stem cells [39]. Breast cancer stem cells, while in a quiescent state, will not respond to drug treatments and may subsequently, following initial remission of the cancer, lead to drug resistance, tumor metastases and progression [40]. All of these factors may influence the efficacy of T-DM1 in addition to the others we have mentioned so far.

We anticipate that drug efficacy studies performed on 3D cultured spheroids and aggregates will become an important biologically relevant model for determining drug activity in tumor tissues. Tumor tissues in vivo contain 3-dimensional structures that affect cellular morphology, surface biomarkers, cell–cell and cell–ECM interactions, and metabolism. Also, the tumor microenvironment plays a major part in cancer. The interaction and communication among cells, as it may be affected by the tumor microenvironment, has been associated with the regulation of tumor growth, metastasis, and even treatment outcome [41]. We understand that our 3D culture model does not replicate all features of in vivo tumors, such as the vasculature or immune response or others that may also be important. However, by adapting 3D cell culture methods and growing organoids (as an extension of the 3D cell culture we performed), we can provide a tool for studying heterogeneity and drug resistance in vitro that is otherwise very difficult and may be impossible to do with 2D cell culture methods.
